# Supplementary figures and images for: Functionally linked amygdala and prefrontal cortical regions are innervated by both single and double projecting cholinergic neurons
Source: Front Cell Neurosci. 2024 Jul 10;18:1426153. doi: 10.3389/fncel.2024.1426153 (PMC11266109; doi:10.3389/fncel.2024.1426153)

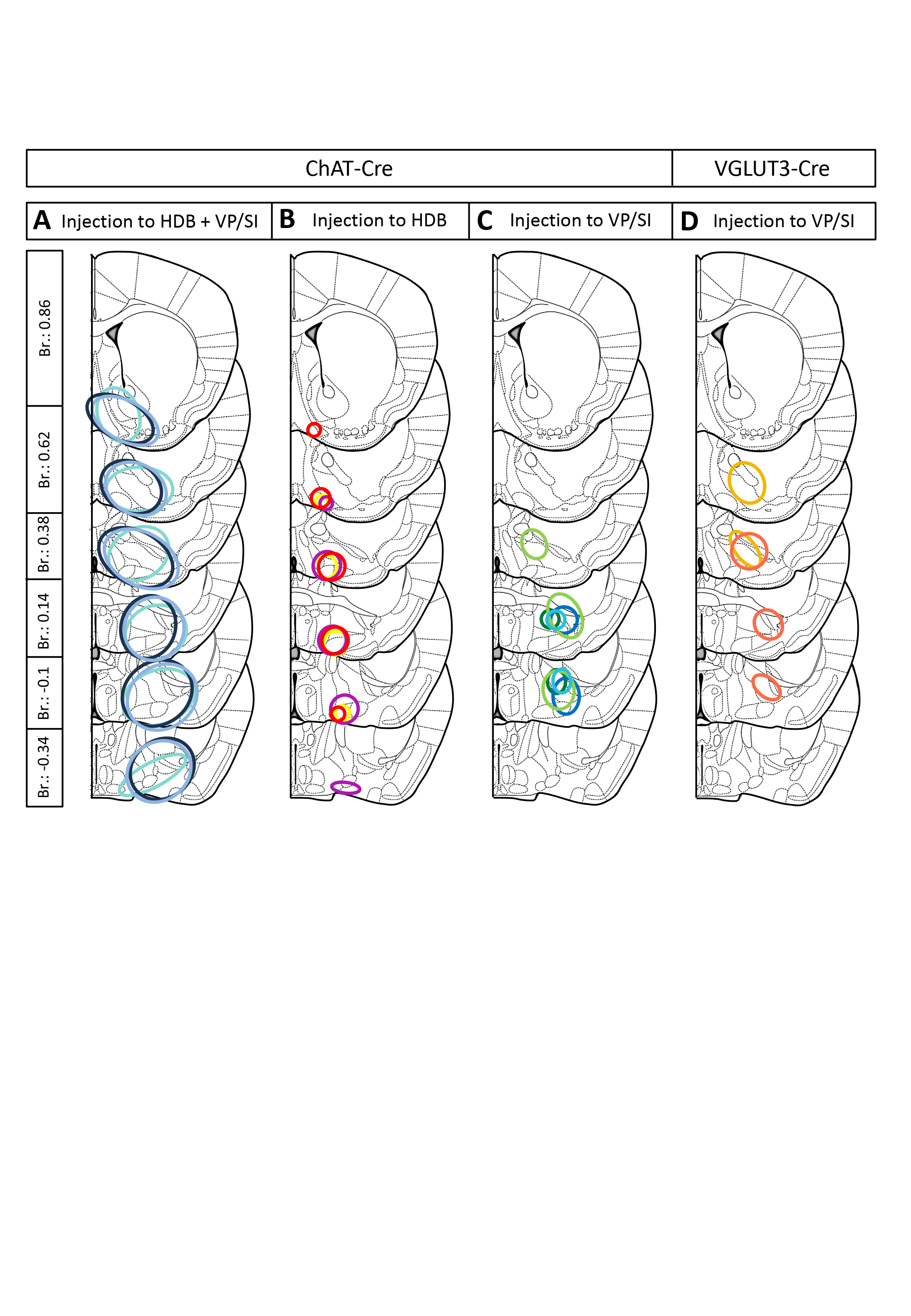

Supplement: SUPPLEMENTARY FIGURE 1 — Reconstructed maps showing the localization and spread of virus injections used for BF anterograde labeling. (A) Localization and spread of 100+100nl AAV5.Ef1a.DIO.eYFP virus injection to the HDB and VP/SI in ChAT-Cre mice (n = 3). Different colors represent different animals. These injections appertain to Figures 1, 4. (B,C) Localization and spread of 30nl AAV8.CAG.flex.GFP virus injected to the HDB (B, n = 3) or VP/SI (C, n = 4) in ChAT-Cre mice. Different colors represent different animals. These injections appertain to Figures 3, 5, and color codes are the same as in the corresponding figures. (D) Localization and spread of 30nl AAV8.CAG.flex.GFP virus in the VP/SI (n = 2) in VGLUT3-Cre mice. Different colors represent different animals. [file Image_1.TIF]

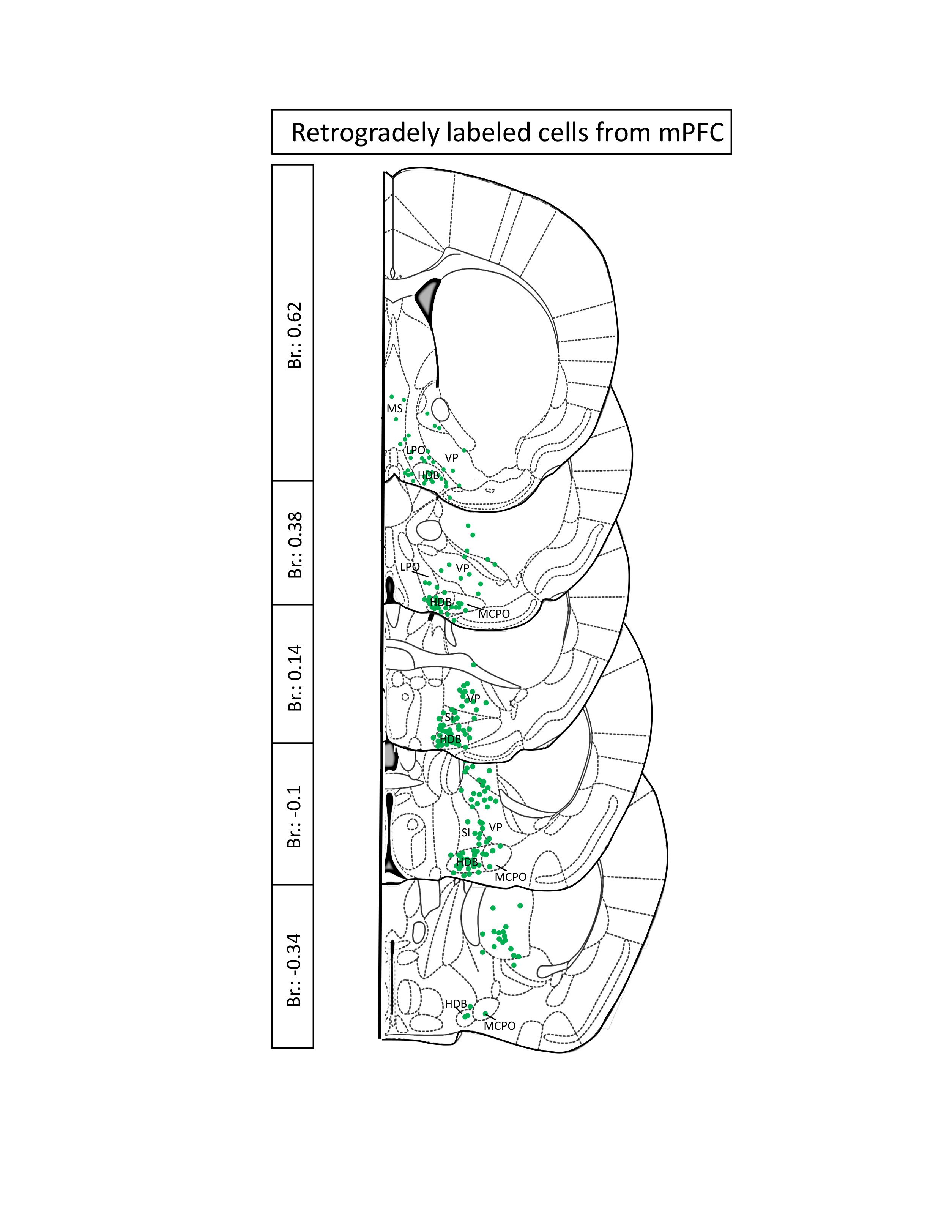

Supplement: SUPPLEMENTARY FIGURE 2 — Localization of retrogradely labeled cholinergic BF neurons from retrograde- anterograde virus tracing. Green dots represent retrogradely labeled BF cholinergic neurons projecting to the mPFC. The area covered by greens dots defines the source of single and double- projecting cholinergic neurons in the BF. Aca, anterior commissure, anterior part; HDB, nucleus of the horizontal limb of the diagonal band; LPO, lateral preoptic area; MCPO, magnocellular preoptic nucleus; MS, medial septal nucleus; SI, substantia innominata; VP, ventral pallidum. [file Image_2.TIF]

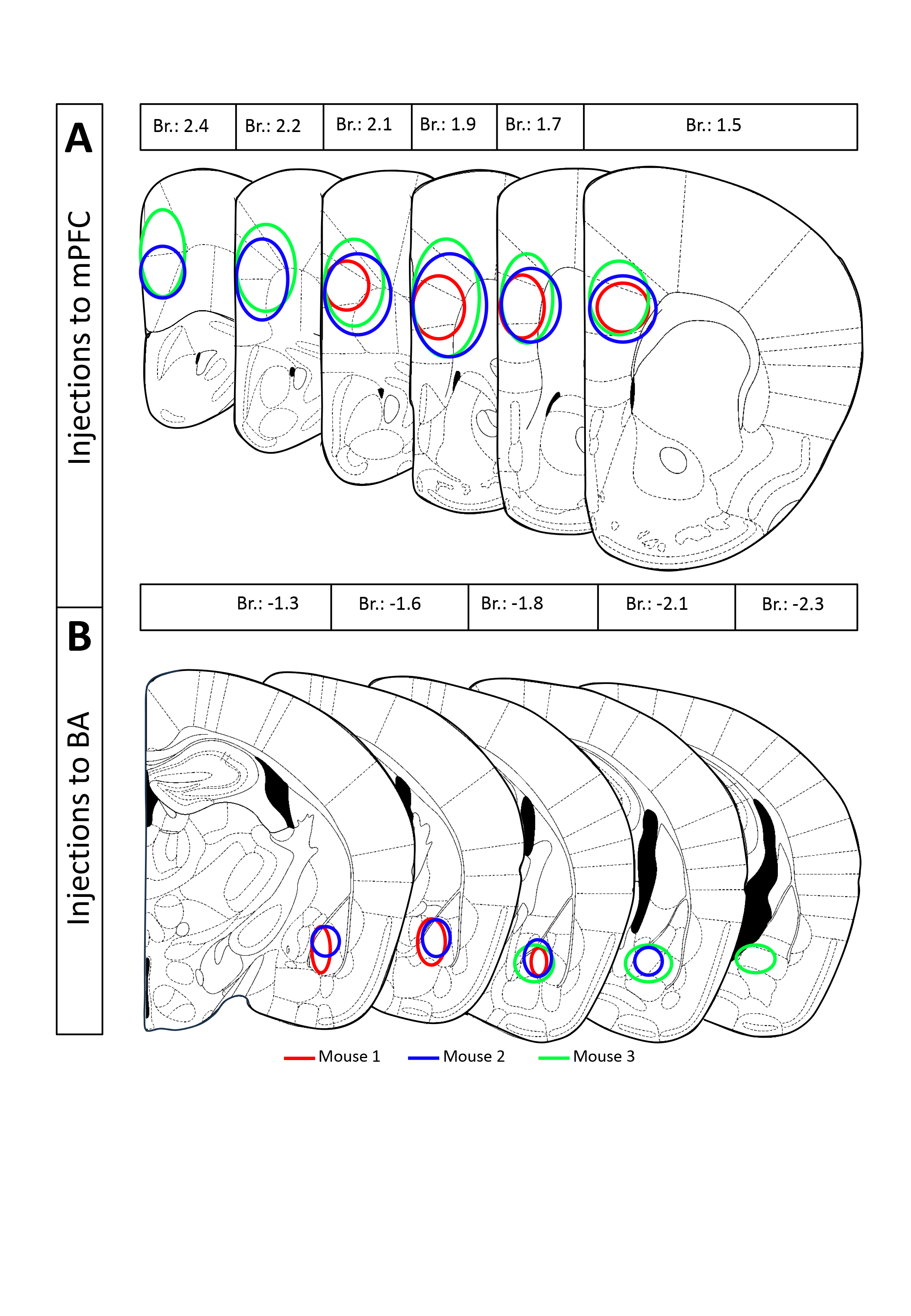

Supplement: SUPPLEMENTARY FIGURE 3 — Reconstructed maps showing the localization and spread of retrograde tracers used for double-retrograde tracing. (A) Localization and spread of Fluorogold in the mPFC (n = 3). (B) Localization and spread of Fast Blue in the BA (n = 3). Different colors represent different animals. [file Image_3.TIF]

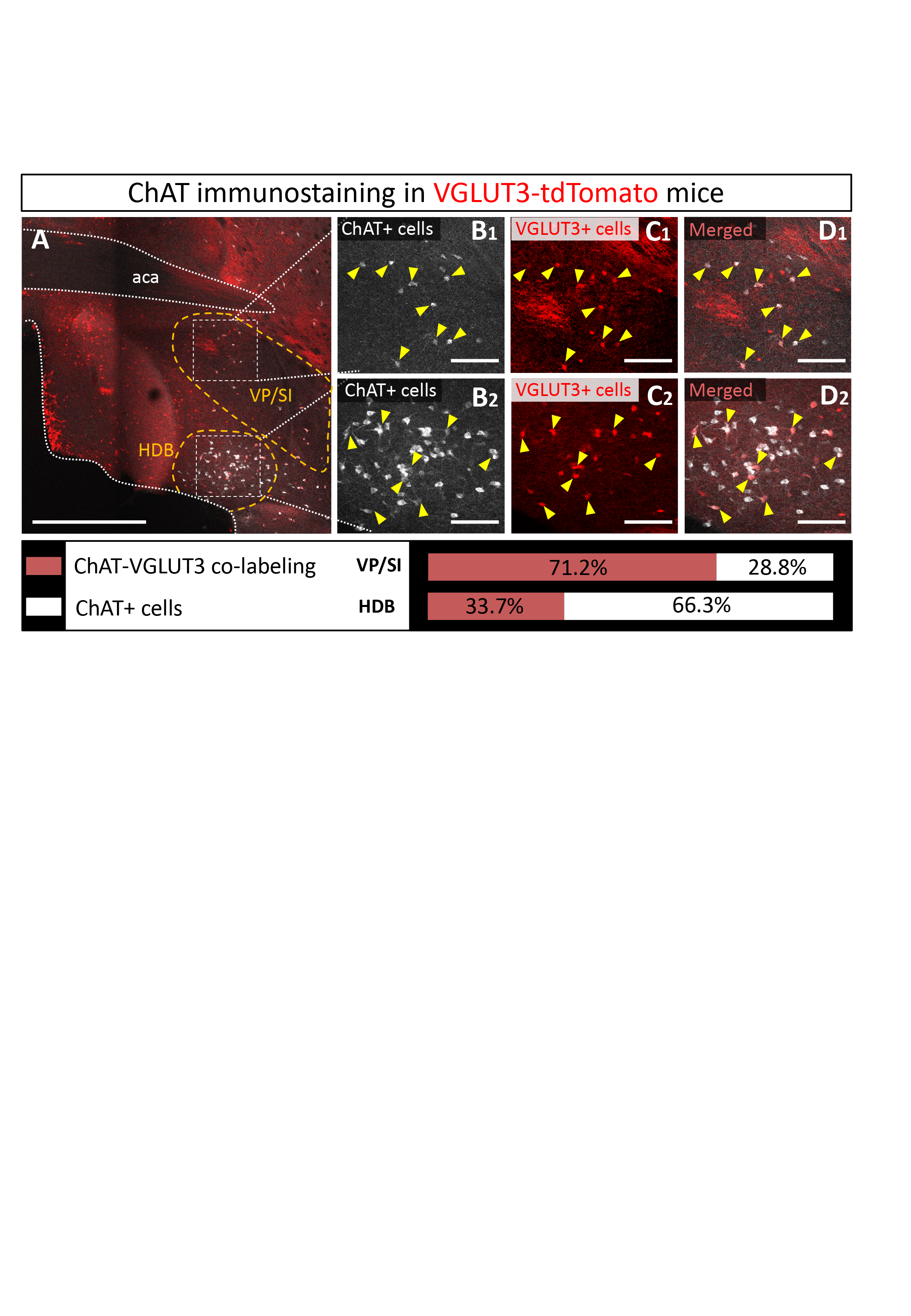

Supplement: SUPPLEMENTARY FIGURE 4 — Ratio of ChAT and VGLUT3 co-labeling in the HDB and VP/SI. (A) Example coronal slice taken at the BF, showing ChAT content of VGLUT3-expressing neurons. The yellow dashed lines represent the borders of the HDB and VP/SI, where the co-labeling was counted. Scale bar: 500 μm. (B) ChAT+ cells in the VP/SI (B1) and HDB (B2). Scale bar: 100 μm. (C) VGLUT3+ cells in the VP/SI (C1) and HDB (C2). Scale bar: 100 μm. (D) Merged images taken at the VP/SI (D1) and HDB (D2). Scale bar: 100 μm. Yellow arrows indicate co-labeled neurons. Bottom graph showing the ratio of co-labeling in the VP/SI and HDB, respectively. [file Image_4.TIF]

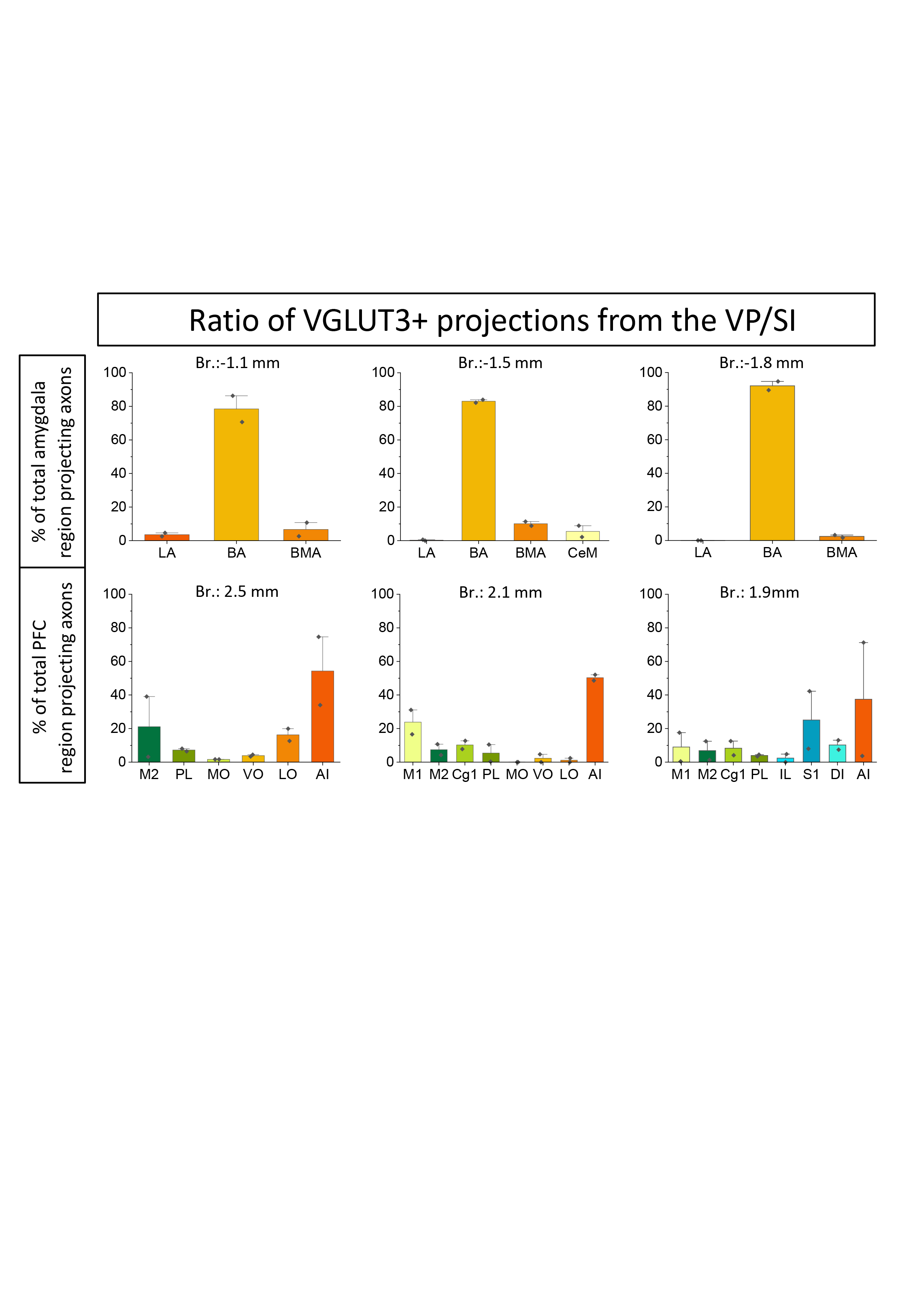

Supplement: SUPPLEMENTARY FIGURE 5 — Ratio of glutamatergic (VGLUT3) projections in the amygdala and PFC subregions originating from the VP/SI. (A) Percentage of amygdala region-projecting VP/SI glutamatergic axons in each nucleus/region at different bregma levels. (B) Percentage of PFC region-projecting VP/SI glutamatergic axons in each nucleus/region at different bregma levels. These graphs appertain to Figure 8. [file Image_5.TIF]
